# Evaluating the impact of prescription drug monitoring program implementation: a scoping review

**DOI:** 10.1186/s12913-017-2354-5

**Published:** 2017-06-20

**Authors:** Erin P. Finley, Ashley Garcia, Kristen Rosen, Don McGeary, Mary Jo Pugh, Jennifer Sharpe Potter

**Affiliations:** 1 University of Texas Health Science Center San Antonio, 7703 Floyd Curl Drive, San Antonio, TX 78229 USA; 2 South Texas Veterans Healthcare System, 7400 Merton Minter Boulevard, San Antonio, TX 78229 USA

**Keywords:** Prescription Monitoring Program, Opioid Risk Mitigation, Scoping Review, Evaluation, Health policy

## Abstract

**Background:**

Prescription drug monitoring programs (PDMPs) have been implemented in 49 out of 50 states in an effort to reduce opioid-related misuse, abuse, and mortality, yet the literature evaluating the impact of PDMP implementation remains limited. We conducted a scoping review to: (1) describe available evidence regarding impact of PDMPs in the U.S.; and (2) propose a conceptual model to inform future PDMP implementation and evaluation efforts.

**Methods:**

Scoping systematic review following Arksey and O’Malley’s (2005) methodology. We identified 11 relevant studies based on inclusion criteria using a PubMed database search of English-language studies published 1/1/2000–5/31/16. Data were extracted and thematic analysis conducted to synthesize results.

**Results:**

Extant evidence for the impact of PDMPs as an opioid risk mitigation tool remains mixed. Thematic analysis revealed four domains of opioid-related outcomes frequently examined in original studies evaluating PDMP implementation: (1) opioid prescribing; (2) opioid diversion and supply; (3) opioid misuse; and (4) opioid-related morbidity and mortality. An evaluation framework incorporating these domains is presented that highlights significant gaps in empirical research across each of these domains.

**Conclusions:**

Evidence for the impact of state-level PDMPs remains mixed. We propose a conceptual model for evaluating PDMP implementation toward the goals of clarifying PDMP mechanisms of impact, identifying characteristics of PDMPs associated with best outcomes, and maximizing the utility of PDMP policy and implementation to reduce opioid-related public health burden.

## Background

Misuse of opioid analgesics is an urgent public health concern. Findings from the 2014 National Survey on Drug Use and Health revealed non-medical use of opioid analgesics is second only to marijuana with respect to illicit substance use [1]. Since the early 90’s, opioid prescribing and dispensing rates have increased, accompanied by an increase in opioid overdose morbidity and mortality rates [2, 3]. As of 2014, 4.3 million individuals within the U.S. reported non-medical use of opioids within the past month [1], and more deaths from drug overdose were recorded than in any previous year. Of the 47,055 overdose deaths that occurred, 61% were caused by prescription opioids [4].

To address the growing problem of opioid misuse and abuse, 49 of 50 states in the U.S. have implemented electronic prescription drug monitoring programs (PDMPs) that track scheduled medications dispensed from pharmacies in an effort to mitigate prescription misuse and diversion, often with financial support from the U.S. Bureau of Justice Assistance [5]. PDMPs require routine, scheduled reporting by pharmacies of prescription-related data for all medications of interest. Typically such information includes medication data for the past year, date medications were dispensed, and information on patient, prescriber, pharmacy, medicine, and dose. Fulfilling both healthcare and legal functions, PDMPs can be used to generate individual-level reports providing a list of all scheduled prescriptions dispensed during a given period of time, or population-level reports identifying broader epidemiologic trends in controlled substance use within and across states. Law enforcement agencies have made use of PDMP data to identify fraudulent prescribing or illegal activity related to diverting controlled substances [6]. Prescribers and pharmacists have access to patients’ medication data and, in some states, unsolicited reports may be delivered if embedded algorithms detect patterns indicating potential misuse, abuse, or diversion [5]. These algorithms vary by state, but may, for example, be triggered when a patient receives scheduled medications from five or more prescribers at five or more pharmacies (“5 × 5″), or three or more early refills within a 3 months period (“3 × 3″) [5].

Although PDMP implementation occurred as early as 1939 in California, and in 1972 in Pennsylvania, many states have initiated PDMPs only within the past decade. In 2001, only 16 had passed legislation regarding the implementation of PDMPs, but by 2012, 49 states had passed similar legislation [5]. Because PDMPs have been adopted at the level of individual states, there is considerable variation in state policy regarding such elements as data reporting, how queries are generated, and the responsibilities of prescribers and law enforcement in prescription monitoring [7]. For example, states such as Delaware, North Dakota, and Utah mandate providers query the PDMP based on subjective “judgment of inappropriate use”, while Oklahoma requires prescribers to check its PDMP only when prescribing, administering, or dispensing methadone [7]. Perhaps as a result of this diversity, relatively little empirical research has examined the impact of PDMPs on opioid-related outcomes of concern, and PDMPs’ effectiveness as an opioid risk mitigation tool remains to be determined. Although selected best practices for PDMPs have been proposed [8], including recommendations that both provider enrollment and utilization be mandated, no standardized model has yet been proposed to facilitate evaluation or comparison of PDMP-related impacts. Defining the desired outcomes of PDMPs and the suspected mechanisms underlying these outcomes is likely to be of value in efforts to improve existing PDMPs and structure those being newly implemented in other systems, particularly in providing a rubric for evaluation that supports valid and reliable assessment of complex PDMP models across diverse settings.

The goals of the current project were therefore to: (1) synthesize the available evidence regarding the impact of PDMP policy and implementation on opioid analgesic misuse within the U.S.; and (2) building upon this literature, to develop a conceptual model in support of future efforts to evaluate PDMP implementation. To these ends, we conducted a scoping review. Scoping reviews are considered preferable to a traditional systematic review when the aim is to “map rapidly the key concepts underpinning a research area and the main sources and types of evidence available” [9]. Scoping reviews are of particular value in enabling synthesis of research that is complex and makes use of a variety of study designs. Because they allow for summary of studies drawing upon diverse outcome measures, as is the case with the emerging literature on PDMPs [10], they can facilitate rapid dissemination of knowledge where the extant research does not yet support systematic review.

## Method

We followed the five steps of the Arksey and O’Malley [9] method for scoping studies (Table 1), which include: 1) identifying one or more research goals; 2) identifying relevant studies; 3) selecting appropriate studies for data extraction; 4) charting the data in selected studies; and 5) collating, summarizing, and reporting results. We began by identifying our primary goal as describing the available evidence regarding impact of PDMPs in the United States. Our secondary objective was to propose a conceptual model for PDMP evaluation to inform implementation and refinement efforts and identify key gaps in existing research.

**Table 1:** Overview of Scoping Review (Adapted from Arksey and O’Malley [9])

Step 1. Identify research goal(s)	1) Describe available evidence regarding impact of PDMPs in the United States; 2) Propose a conceptual model for PDMP evaluation to inform future implementation and evaluation efforts.
Step 2. Identify relevant studies	PubMed database search of English-language studies published between 1/1/2000–08/18/2015 using key words “Prescription drug monitoring program” and “Opioid prescription monitoring program”. Inclusion criteria: human; English language; original research; peer-reviewed; direct assessment of outcomes related to impact or effectiveness of PDMP implementation. Additional studies were identified using reference lists of relevant articles. Prior to submission, this search was updated to include articles from 8/19/2015–5/31/16.
Step 3. Study selection	Irrelevant and duplicate articles were identified by two reviewers at the abstract and title level. Systematic reviews, commentaries, and non-U.S. studies were excluded. Full text of original studies remaining was examined by two reviewers. A third reviewer provided input as needed to achieve consensus.
Step 4. Charting the data	A data chart collection form was developed to facilitate extraction of findings and key contextual factors from each study. Consistent with scoping methodology, this sheet was updated collectively and iteratively as familiarity with literature increased. Two reviewers independently assessed articles, then met to determine compatibility in approaches. A third reviewer provided input when needed to achieve consensus.
Step 5: Collating, summarizing, and reporting results	The authorship team then independently and collaboratively reviewed summative findings of the data extract, resulting in: identification of distinct domains of opioid-related outcomes hypothesized to be associated with PDMP implementation; conceptualization of an evaluation framework; and synthesis of current PDMP research findings, including recognition of research gaps.

### Search strategy

**Fig. 1:**
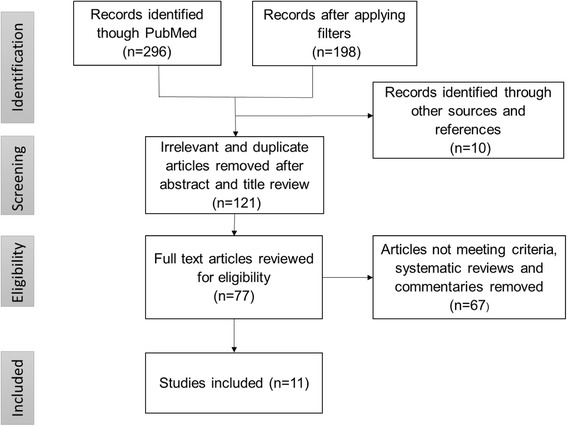
Article Identification and Selection Process (Adapted from PRISMA (Moher, [24])). *Figure Note: Prior to submission, an updated search for newly published research through 05/31/2016 was conducted by the authors using the same strategy for search and review, resulting in the addition of one article meeting inclusion criteria. The final number of articles included was 11

An initial PubMed database search for English-language peer-reviewed articles was carried out using the key words “prescription drug monitoring program” and “opioid prescription monitoring program”. Non-human studies were eliminated through filtering. As noted above, the majority of PDMPs proliferated between 2001 and 2012; to ensure inclusion of relevant studies, we included articles published between 1/1/2000 and 08/18/2015. One hundred twenty-one relevant articles were identified using this approach. An additional 10 studies were identified from review of article references from the initial search. Publications were then reviewed to assess for inclusion criteria defined a priori, specifically: peer-reviewed; presents original research; provides direct assessment of outcomes related to impact or effectiveness of PDMP implementation. Following this process (summarized in Fig. 1), 10 articles remained for review and synthesis. Prior to submission, an updated search for newly published research through 05/31/2016 was conducted by the authors using the same strategy for search and review, resulting in the addition of one article meeting inclusion criteria. The final number of articles included was 11.

### Data synthesis and analysis

For the purpose of understanding key concepts and sources of evidence in this literature, thematic analysis was conducted following article review in the course of meetings by the research team, and a data chart developed for extracting findings and key contextual indicators. Each article was reviewed for data extraction by two members of the research team, with discrepancies resolved through discussion and consensus in meetings with a third reviewer and ultimately, with the full authorship team. The authorship team then independently and collaboratively reviewed summative findings of the data extract, resulting in: identification of distinct domains of opioid-related outcomes hypothesized to be associated with PDMP implementation; conceptualization of an evaluation framework building upon these domains; and synthesis of current PDMP research findings, including recognition of research gaps.

## Results

### Thematic analysis

**Fig. 2:**
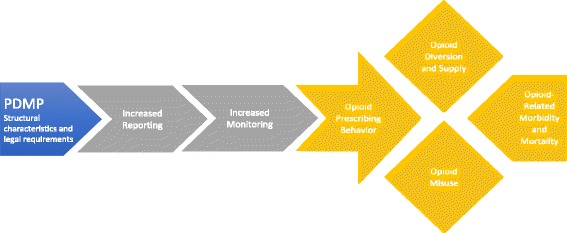
Conceptual Model of the Impact of Prescription Drug Monitoring Programs

Thematic analysis revealed that studies of PDMPs generally emphasize an underlying link between opioid prescription monitoring and prescribing, on the one hand, and misuse, diversion, and morbidity/mortality, on the other. Studies anticipate that the increased monitoring and tracking of prescription drugs supported by PDMPs will facilitate reporting in two ways: first, by generating reports for providers that detail a patient’s medication history and previous prescriptions; and second, by identifying potential indications of drug abuse or diversion using algorithms or “risk triggers”, such as patients with 5 prescribers and 5 pharmacies in a 3 month period [5]. Providers informed in this way regarding a patient’s history and likely signs of misuse or diversion are expected to reduce or refine their opioid prescribing, thus decreasing misuse and diversion of prescription opioids and consequently mortality and morbidity rates. The logic of PDMP evaluation studies, therefore, consistently presumes that increased monitoring and reporting of opioid prescriptions will be associated with changes in opioid-related outcomes across one or more domains: 1) opioid prescribing behavior, e.g., a reduction in opioid prescribing; 2) opioid diversion and supply; 3) opioid misuse; e.g., doctor shopping; and 4) opioid-related morbidity/mortality, e.g., substance use disorder or overdose. An additional finding of thematic analysis was that the impacts of PDMP implementation must be considered in terms of both potential benefits and potential harms, particularly related to findings in the domains of prescribing behavior and morbidity/mortality. For example, considerable concern has been expressed about the potential for a “chilling effect” [11, 12] of PDMPs on providers’ opioid prescribing that might deprive patients of adequate pain control [13], and unclear long-term unintended consequences [14]. The resulting conceptual framework for PDMP evaluation is depicted in Fig. 2.

### Research findings

Table 2 provides a summary of articles addressing the impact of PDMPs. Following scoping methodology [9], data were charted to extract key designs and findings identified in the literature, organized as follows: author and year; study design; outcome measure(s); study findings; and whether the study provides evidence to support beneficial PDMP impact and in which domain(s) (i.e., opioid prescribing behavior, opioid diversion and supply, opioid misuse, and/or opioid-related morbidity/mortality).

**Table 2:** Studies of Prescription Drug Monitoring Program (PDMP) Impact by Domain of Opioid-Related Outcome Measure

Article	State(s)/Years Examined	Outcome measure	Design/Methods	Findings	Evidence for PDMP Benefit
Domain 1: Opioid Prescribing Behavior					
Paulozzi, 2011^a^ [3]	PDMP and non-PDMP states; 1995–2005	Mean MME rates	Crude mean MME^b^ rates and their standard errors for PDMP and non-PDMP states were calculated by year and across 1999–2005 timespan.	According to results of a regression analysis, the presence of a PDMP was not a significant predictor of MME rates.	No
Brady, 2014 [2]	PDMP and non-PDMP states; 1999–2008	Opioids dispensed per quarter for each state from 1999 to 2008	Multivariable linear regression model with generalized estimating equations assessed the effect of state PDMPs on per-capita dispensing of MMEs.	Overall, implementation of state PDMPs up to 2008 did not show significant impact on per-capita opioids dispensed. Examined state-by-state, authors found PDMP implementation associated with per capita MME decline in 9 states, increase in 8 states, and no effect in 14 states.	No
Rasubala, 2015 [21]	New York; 2012–2014	Frequency and volume of opioid prescriptions by dentists in a dental urgent care center	Cross-sectional survey of a dental urgent care center 3 months before and 6 months after implementation of a PDMP	Total prescribed opioids decreased 78% by dentists in a dental urgent care center after a mandatory PDMP was implemented.	Yes
Ringwalt, 2015 [11]	North Carolina; 2009–2011	Number of filled prescriptions for opioids	Examined associations between total number of providers who used the PDMP, mean number of days providers queried the system, and filled opioid prescriptions.	Strong positive association between increasing use of PDMP and opioid analgesic prescriptions over time.	No
Rutkow, 2015 [25]	Florida; 2010–2012	Opioid volume, per transaction, MME prescribed, MME per transaction, days’ supply per transaction, prescriptions dispensed.	Comparative interrupted time-series analysis to assess the effect of PDMP and ‘pill mill law’ implementation on a closed cohort of prescribers, retail pharmacies, and patients.	Jointly the PDMP and ‘pill mill’ policies were associated with reductions in total opioid volume, mean MME per transaction, and total number of opioid prescriptions dispensed.	Yes
Domain 2: Opioid Diversion and Supply					
Reisman, 2009^a^ [12]	PDMP and non-PDMP states; 1997–2003	State prescription opioid shipments (ARCOS)^b^	Compared state prescription opioid shipments in 14 states with PDMPs (intervention group) and 36 states without PDMPs (control group).	States with PDMPs received fewer oxycodone shipments that non-PDMP states; opioid shipments in all states continued to rise.	Yes
Surratt, 2014 [26]	Florida; 2009–2012	Quarterly prescription opioid diversion rates	Changes in prescription opioid diversion rates identified using quarterly law enforcement data after implementation of PDMP and ‘pill mill’ laws assessed using hierarchical linear models.	Significant decline in oxycodone diversion; nonsignificant (*p* = 0.08) decline in hydrocodone diversion; no decline in fentanyl, hydromorphone, or tramadol.	Yes
Domain 3: Opioid Misuse					
Reifler, 2012^a^ [15]	PDMP and non-PDMP states; 2003–2009	Cases of intentional exposure to opioids (RADARS)^b^	Repeated measures negative binomial regression was applied to quarterly case data to estimate opioid misuse trends. PMP presence was modeled as a time-varying covariate for each state.	Results suggest PDMPs are associated with a mitigation of increasing opioid misuse over time in both the general population as well as within the population seeking treatment at Opioid Treatment Programs.	Yes
Domain 4: Opioid-related Morbidity/Mortality					
Reisman, 2009^a^ [12]	PDMP vs. non-PDMP states; 1997–2003	Inpatient prescription opioid treatment admissions per year	Inpatient admissions for prescription opioid abuse (TEDS)^b^ in 14 states with PDMPs (intervention group) and 36 states without PDMPs (control group).	PDMP states reported a smaller increase in opioid treatment admissions per year (p[=0.06). Patients receiving inpatient drug treatment in PDMP states were less likely to have been admitted for prescription opioids.	Yes
Paulozzi, 2011^a^ [3]	PDMP and non-PDMP states; 1999–2005	Rates of drug overdose and opioid-related mortality by state	Regression analysis using mortality data by state and year, crude mean mortality and standard error for PDMP and non-PDMP states.	Mortality rates did not differ by a statistically significant margin between PDMP and non-PDMP states.	No
Reifler, 2012^a^ [15]	PDMP and non-PDMP states; 2003–2009	Opioid treatment admissions	Repeated measures negative binomial regression applied to quarterly surveillance data from 2003 to mid-2009 to estimate opioid abuse trends. PDMP presence was modeled as a time-varying covariate for each state.	States with PDMPs appeared to experience smaller increases in drug abuse over time.	Yes
Li, 2014 [16]	PDMP and non-PDMP states; 1999–2008	Drug overdose mortality data for state-quarters	Multivariate negative binomial regression modeling examined drug overdose mortality for states with and without PDMPs during 1999–2008.	PDMP states experienced higher drug overdose mortality overall; PDMP impact on mortality varied by state.	No
Delcher, 2015 [17]	Florida; 2003–2012	Monthly counts of oxycodone-caused deaths	Time-series, quasi-experimental research design with ARIMA^b^ statistical models examined monthly counts of oxycodone-caused deaths using a binary variable (pre/post-implementation).	Implementation of Florida’s Prescription Drug Monitoring Program was associated with a significant decline in oxycodone-caused mortality	Yes
Maughan, 2015 [27]	11 Multi-state metropolitan areas; 2004–2011	Rates of emergency department visits involving opioid analgesics	Using retrospective data (DAWN)^b^, generalized estimating equations assessed PDMP implementation and opioid-related morbidity.	PDMP implementation was not associated with change in rates of ED visits involving opioid analgesics.	No

^a^Article findings addressed more than one domain of opioid-related outcome

^b^ *MME* Morphine Milligram Equivalents, *ARCOS* Automation of Reports and Consolidated Orders Systems, *RADARS* Researched, Abuse, Diversion and Addiction-Related Surveillance system, *TEDS* Treatment Episode Data Sets, *ARIMA* Autoregressive Integrated Moving Average models, *DAWN* Drug Abuse Warning Network

As Table 2 makes clear, the extant literature reveals mixed findings about the impact of PDMPs as a tool for reducing misuse and diversion of controlled substances. There is evidence for reduced opioid prescribing following implementation of a PDMP in studies conducted in Florida and New York, but no significant trend emerges in similar studies conducted in North Carolina or combining results from multiple PDMP and non-PDMP states. Studies of opioid diversion and supply found evidence for reduced shipments of oxycodone in PDMP states, but no reduction of opioid shipments overall. A study of diversion in a single state, Florida, found significant reductions in diversion of oxycodone, morphine, and methadone, but not in hydrocodone, fentanyl, or tramadol. The single study identified that examined the association between PDMP implementation and patterns of opioid misuse directly found evidence that the presence of a PDMP helped to slow the increase in rates of misuse, but did not achieve reductions in misuse overall. Studies of opioid-related morbidity and mortality found smaller increases in opioid treatment admissions in PDMP than non-PDMP states [12, 15], but no clear pattern of reduced overdose mortality in PDMP states overall [4, 16]. Mortality rates did appear to be lower in the specific PDMP states of California, Texas, and New York [3], and there was an immediate drop in mortality following PDMP implementation in Florida [17].

## Discussion

A critical finding of this synthesis has been that studies of opioid-related outcomes associated with PDMP implementation typically point to a shared logic for how PDMPs are expected to function, namely that: implementation of PDMPs will increase reporting and monitoring of controlled prescriptions, resulting in reduced opioid prescribing by providers, reduced opportunities for opioid diversion and misuse, and lower frequency of negative consequences such as opioid abuse and mortality [18]. Despite this shared logic, however, there is a marked lack of discussion in the literature to date regarding the scope of PDMP-related outcomes that should be examined and assessed in order to evaluate whether, and under what conditions, their implementation is having the intended impact.

In conducting this review, therefore, we found it useful to identify four domains of opioid-related outcomes frequently examined in original studies evaluating PDMP impacts: opioid prescribing; opioid diversion and supply; opioid misuse; and opioid-related morbidity and mortality. While these domains are subject to debate and may at times overlap, we believe they provide a useful heuristic for identifying areas of relative strength and weakness in the existing evidence for the impact of PDMPs.

While the literature evaluating PDMPs remains relatively nascent, a complex picture is emerging. Studies examining the association between PDMP implementation and opioid-related outcomes do not indicate a consistent pattern of discernible change. Such variation in results is likely due in part to variation in study-related factors, including study design and methods, use of inconsistent measures of impact, and examination of PDMP impacts in a single state vs. across multiple states. Additionally, the characteristics of PDMPs themselves vary considerably across states in both legislated components and strategies for implementation. Use of PDMPs by providers prior to writing a prescription for opioids may be mandatory or optional, and states vary in the responsibility they place upon providers for any negative outcomes associated with misuse or abuse by their patients [5]. PDMPs also vary in the frequency with which data is reported to them by participating pharmacies, the ease of accessing necessary information, the types of providers allowed to register, the information available, the amount of training providers receive in use of PDMPs, and by which state agencies they are administered [5]. As a result, the timeliness and accuracy of PDMP data varies considerably across states, as does the frequency and consistency of use by providers. It was unsurprising to find two studies examining the impact of PDMP implementation on opioid diversion, given the important role played by the Bureau of Justice Assistance in supporting PDMP implementation [8]. However, reviewing the evidence makes it clear that more nuanced investigation of the impact of specific characteristics of PDMP legislation and implementation will be necessary to firmly establish the policy features and strategies associated with PDMPs that are successful in reducing negative outcomes as intended.

Even within the limitations of the current evidence, however, it has already become clear that PDMPs may also be associated with impacts beyond those generally hypothesized, both potential benefits and harms. Studies have reported that many clinicians find PDMPs useful as a tool for communication and interaction with patients [19, 20]. With patient prescription history at their disposal, providers can not only verify the patient’s current prescriptions to avoid doctor shopping or drug abuse, but can also avoid potentially dangerous non-controlled drug interactions. As noted above, an important concern has been raised regarding the “chilling effect” that PDMPs and other opioid control measures may have on providers’ opioid prescribing, leaving patients potentially undertreated for pain or seeking elsewhere for licit or illicit means to manage their pain [11]. What happens when providers re-evaluate their opioid prescribing has proven to be a critical question, although relatively few studies have yet provided data to answer it. Of the studies examined in this review, Rasubula et al. [21] found that dentists reducing their prescriptions of opioid analgesics in a dental urgent care center correspondingly increased their use of non-opioid analgesics, such as acetaminophen, and in this case drew closer to recommended practice guidelines for post-operative management of oral pain. Paulozzi et al. [3]’s findings of increased prescribing of hydrocodone, then a Schedule III drug, in PDMP states may also indicate that some providers have responded to PDMPs and associated shifts in prescribing norms by increasing prescriptions of analgesics from lower schedules. More troublingly, there is also evidence that patients, when faced with reduced ability to access licit opioids, may turn to illicit heroin, morphine, or fentanyl as alternatives, with studies indicating an increase in related mortality in some PDMP states [17, 22, 23].

There are several limitations to this review. Because the PDMP literature remains small and study outcomes and design vary, we were unable to conduct a traditional systematic review or meta-analysis, thus limiting our ability to conduct statistical analysis of the cumulative evidence. Because we described state-administered PDMP programs exclusively, findings may not extend to other prescription monitoring approaches in the U.S. and elsewhere. Nonetheless, this scoping review may inform other monitoring efforts, particularly by underscoring the importance of having clearly defined target outcomes (e.g., reduction in opioid-related morbidity and mortality) and a plan for evaluation. Conclusive evidence regarding impact cannot be determined from observational/cross sectional designs, and data to support causal relationships between PDMP implementation and opioid-related outcomes remain limited as a result. Drawing upon PubMed as the core search database may have resulted in identifying more literature emphasizing healthcare policy rather than law enforcement impacts of PDMPs. In addition, this review was limited to published data; additional analyses may be available in unpublished reports from state or other sources, and should be considered for inclusion in future systematic reviews.

## Conclusions

We believe the conceptual framework and synthesis of findings presented here offer valuable tools for evaluating the body of knowledge around PDMPs as policy and research in this area continue to progress. Establishing a conceptual framework for PDMP evaluation is helpful in clarifying areas of relative strength and weakness in the literature. For example, we identified only a single study examining opioid misuse as an outcome of PDMP implementation [15], a concerning gap given the level of national concern about opioid misuse and its potential consequences for leading to abuse and/or overdose. Moreover, evaluating the literature available along each step of the conceptual framework makes it clear how poorly we yet understand the real-time consequences of PDMP implementation, or the nuances of how specific characteristics of PDMP policy or implementation may impact downstream effects. More sophisticated analysis of specific components of PDMPs will be required to fully understand widely varying impacts across states.

Although PDMP implementation has been initiated across the United States, little consistent evidence has yet emerged to demonstrate PDMPs’ impact on outcomes of greatest importance, whether more proximal targets such as prescribing behavior or distal outcomes such as opioid misuse, diversion, morbidity and mortality. We offer a call to action to engage in rigorous examination of PDMP impacts across the range of domains identified here, and particularly with regard to opioid misuse, and to do so with a careful eye to understanding features of PDMP legislation and implementation associated with positive outcomes. This call comes at a time when the field of PDMP evaluation is rapidly maturing and more information is becoming available through data sharing and linking with electronic medical records. The increased analytic capacity enabled by such growth should directly facilitate the examination of algorithms for identifying opioid prescribing, misuse, and abuse that are so much a part of the promise of PDMPs, but which have not yet achieved their full potential in mitigating opioid-related harms for individuals and populations.

## Abbreviations

PDMPs: Prescription drug monitoring programs
PRISMA: Preferred Reporting Items for Systematic Reviews and Meta-Analyses
U.S.: United States
